# IMPROVED NALOXONE PRESCRIBING IN RURAL PRIMARY CARE FOLLOWING ACADEMIC DETAILING FOR OPIOID OVERDOSE PREVENTION

**DOI:** 10.1093/geroni/igad104.2517

**Published:** 2023-12-21

**Authors:** Leah Tobey, Meghan Breckling, Mohab Ali, Robin McAtee

**Affiliations:** University of Arkansas for Medical Sciences, Little Rock, Arkansas, United States; University of Arkansas for Medical Sciences, Little Rock, Arkansas, United States; University of Arkansas for Medical Sciences, Little Rock, Arkansas, United States; University of Arkansas for Medical Sciences, Little Rock, Arkansas, United States

## Abstract

Performing opioid misuse screenings for older adults (OA) and reviewing high-risk medications are reliable screenings in preventing harm, especially accidental overdose. As one of the HRSA funded Geriatric Workforce Enhancement recipients, the AR Geriatric Education Collaborative (AGEC) worked with a rural federally qualified healthcare clinic system (ARcare) to reduce the likelihood of patient’s accidental overdose by co-prescribing Naloxone, when indicated. An Academic Detailing (AD) team of a PT, PharmD and MD, led this interactive training. AD is clinician-to-clinician support customized with the purpose to connect primary care providers to the best available evidence and most up-to-date clinical practice guidelines. A conversational needs assessment was stimulated where the provider shared their perspectives, knowledge deficits, and needs surrounding the AD topic of Naloxone. These AD interactions encouraged safer prescribing of high-risk medications while considering the use of hard reduction resources for patients, such as prescribing Naloxone. ARcare providers (n=22) attended AD on Preventing Opioid Overdose with Naloxone over 11 months. Of those, 9 had previously prescribed Naloxone 40 times pre-training then 107 times post-training; five providers had never prescribed Naloxone but prescribed it 46 times post-training. Eight providers, made no change, some of whom work in schools. Post-training data gathered from the 14 Naloxone prescribing providers showed a total of 153 Naloxone prescriptions after AD, a 283% increase. For primary care settings, age-friendly medication practices are critically important for OA safety, aging in place, and for OA and their caregivers to understand and utilize Naloxone if accidental overdose is suspected.

